# Culture Change Initiatives in High Medicaid Nursing Homes: Does Time of Adoption Make a Difference?

**DOI:** 10.1093/geroni/igaa057.072

**Published:** 2020-12-16

**Authors:** Latarsha Chisholm, Akbar Ghiasi, Justin Lord, Robert Weech-Maldonado

**Affiliations:** 1 University of Central Florida, Orlando, Florida, United States; 2 University of Incarnate Word, San Antonio, Texas, United States; 3 LSU-Shreveport, Shreveport, Louisiana, United States; 4 UAB, Birmingham, Alabama, United States

## Abstract

Racial/ethnic disparities have been well documented in long-term care literature. As the population ages and becomes more diverse over time, it is essential to identify mechanisms that may eliminate or mitigate racial/ethnic disparities. Culture change is a movement to transition nursing homes to more home-like environments. The literature on culture change initiatives and quality has been mixed, with little to no literature on the use of culture change initiatives in high Medicaid nursing homes and quality. The purpose of this study was to examine how the involvement of culture change initiatives among high Medicaid facilities was associated with nursing home quality. The study relied on both survey and secondary nursing home data for the years 2017-2018. The sample included high Medicaid (85% or higher) nursing homes. The outcome of interest was the overall nursing home star rating obtained from the Nursing Home Compare Five-Star Quality Rating System. The primary independent variable of interest was the years of involvement in culture change initiatives among nursing homes, which was obtained from the nursing home administrator survey. The final model consisted of an ordinal logistic regression with state-level fixed effects. High-Medicaid nursing homes with six or more years in culture change initiatives had higher odds of having a higher star rating, while facilities with one year or less had significantly lower odds of having a higher star rating. Culture change initiatives may require some time to effectively implement, but these initiatives are potential mechanisms to improve quality in high Medicaid nursing homes.

